# Clinical Medicine

**Published:** 1889-12

**Authors:** G. W. Sherbino

**Affiliations:** Abilene, Texas


### CLINICAL MEDICINE.

G. W. Sherbino, M. D., Abilene, Texas.

I. A lawyer was walking along the street one morning when he stopped to show me a sore finger. It proved to be a bone felon; he was predisposed to felons. The end of the thumb was deformed from necrosed bone caused by a felon. His symptoms called for Calc. carb.$^{2c}$. It cured the felon and he never had any more of them. I made a convert of him.

II. A farmer suffering from felon came twelve miles from the country to see me one Saturday, in the fall of 1883. He had suffered excruciating pain for nine days and nights, so that he could not sleep. The finger had been opened and it was discharging ichorous pus, with a large amount of proud flesh, which he had tried to burn off with Alum. But the proud flesh would come back again; the finger now was tied up in tobacco leaves.

Subjective symptoms: Burning at the end of the finger like coals of fire. Aggravation at night, especially after midnight. Sensation of hot needles piercing. Amelioration by hot applications. Arsen.$^{200}$ cured in a day or two; the pain was better in a few minutes.

He wanted to know if I was a faith doctor. I replied, "I have faith in my medicine."

III. A lady complained of pain in the end of her finger like a brier sticking there. The pain got no better from the remedies given. There were redness and swelling, so painful she had to desist from work. The pain then became burning and stinging, better immersed in cold water. I gave a dose of Apis$^{1M}$, waited twenty-four hours. No change, swelling and pain increasing. Hering says if Apis fails then give Sulph. high. At night she got a dose 55M (F.). Next morning pain had all gone; finger not swelled so much. No more medicine, cured promptly.

IV. A carpenter accidentally hit his thumb with a hammer; he suffered with it all night; came to me the next morning. Two doses of Ledum$^{2000}$ cured him.

V. A stock-man was riding through a pasture where there were many mesquit trees, when he stuck a thorn into his second finger from which he suffered for several days. The finger was swelled its whole length, and was stiff and sore. The pain was deep in the bone. Every one who saw it pronounced it a felon. Two doses of Ledum$^{2000}$ cured him, and he did not like to have it split, as was ordered by an allopath.

VI. A clerk in a store came to me with a "sore" on his finger, which had kept him awake for over a week. An opening was at the end, proud flesh protruding. He complained of aching in the arm and hand up to his shoulder, deep in the bone. This kept him awake at night. His pulse was 120; temperature 100°. The pain and the fever were controlled by a remedy having his concomitants in its pathogenesis; but the proud flesh did not yield. I gave a dose of Silicea$^{cm}$ (F.) and Sac. lac. The finger straightway began to discharge laudable pus. He showed the finger to several of his friends who were kind enough to say he would have to have it "split;" it would never get well without. I remarked I would split it from the inside with the dynamic-lance. After his finger was well he again showed it to his friends, who then all agreed with one acclaim it was not a felon.

These cases could be enlarged upon, but enough has been said to show the power of the similars, without antiseptic dressing or the injection of Carbolic acid—the Pennsylvania State Homœopathic Transactions to the contrary notwthstanding. Let such treatment be relegated to eclectics and the allopaths, to whom it rightfully belongs, and not try to palm it off on the public as Homœopathy.

